# Glucose Sensor Design Based on Monte Carlo Simulation

**DOI:** 10.3390/bios15010017

**Published:** 2025-01-04

**Authors:** Gang Xue, Ruiping Zhang, Yihao Chen, Wei Xu, Changxing Zhang

**Affiliations:** 1 Faculty of Civil Engineering and Mechanics, Kunming University of Science and Technology, Kunming 650500, China; xuegang@stu.kust.edu.cn (G.X.); xuwei@kust.edu.cn (W.X.); 2 Intelligent Infrastructure Operation and Maintenance Technology Innovation Team of Yunnan Provincial Department of Education, Kunming University of Science and Technology, Kunming 650500, China; 3 Laboratory of Flexible Electronics Technology, Tsinghua University, Beijing 100084, China; zhangruiping97@foxmail.com (R.Z.); chenyihao92@mail.tsinghua.edu.cn (Y.C.); 4 Key Laboratory of Applied Mechanics, Department of Engineering Mechanics, Tsinghua University, Beijing 100084, China

**Keywords:** Monte Carlo simulation, glucose sensor, biosensor, real-time monitoring

## Abstract

Continuous glucose monitoring based on the minimally invasive implantation of glucose sensor is characterized by high accuracy and good stability. At present, glucose concentration monitoring based on fluorescent glucose capsule sensor is a new development trend. In this paper, we design a fluorescent glucose capsule sensor with a design optimization study. The motion trajectory of incident light in the fluorescent gel layer is simulated based on the Monte Carlo method, and the cloud maps of light intensity with the light intensity distribution at the light-receiving layer are plotted. Altering the density of fluorescent molecules, varying the thickness of tissue layers, and adjusting the angle of incidence deflection, the study investigates the influence of these parameter changes on the optimal position of reflected light at the bottom. Finally, the simulation results were utilized to design and fabricate a fluorescent glucose capsule sensor. Rabbit subcutaneous tissue glucose level tests and real-time glucose solution concentration monitoring experiments were performed. This work contributes to the real-time monitoring of glucose levels and opens up new avenues for research on fabricating glucose sensors.

## 1. Introduction

Diabetes is a chronic disease caused by the pancreas’ insufficient ability to secrete insulin or the body’s failure to effectively utilize the secreted insulin, affecting more than 537 million people worldwide. In 2023, approximately 6.7 million adults died from diabetes or its complications, making it the seventh most common cause of death worldwide. Therefore, self-monitoring blood glucose and thus maintaining normal blood glucose levels is essential to prevent diabetic complications of the heart, kidneys, retina, and nervous system [1]. Most devices on the market today use an invasive fingertip blood collection method to measure glucose concentration in the blood. However, this method can only detect the value of glucose concentration at a certain moment in time and cannot measure its changes. Thus, it is not conducive to the real-time monitoring of blood glucose changes. Moreover, invasive measurements also tend to cause pain and discomfort to the patient during the measurement and even raise the risk of infection [2,3]. In addition to invasive methods of measuring blood glucose, research has shown that non-invasive methods can be used to test blood glucose. The main methods are near-infrared absorption spectroscopy [4,5], mid-infrared absorption spectroscopy [6,7], polarized optical spinning [8,9], Raman spectroscopy [10], and photoacoustic spectroscopy [11]. However, these methods are susceptible to interference by changes in the external environment as well as being affected by differences in the human body, resulting in less accurate test results.

Minimally invasive blood glucose testing methods are characterized by low blood collection, small incisions, and high accuracy [12,13]. Continuous glucose monitoring (CGM) using a glucose sensor allows diabetic patients to measure changes in blood glucose concentration easily and warns the patient if the blood glucose concentration is below or above normal. This method has become a new trend in blood glucose concentration monitoring. Conventional glucose sensors use enzymatic electrochemical methods. However, the presence of enzymes makes it easy to interfere with ambient temperature and other substances, which makes it difficult to adapt to continuous and accurate monitoring scenarios. Therefore, enzyme-free fluorescence-based detection methods have recently become a research hotspot [14,15].

**Figure 1:**
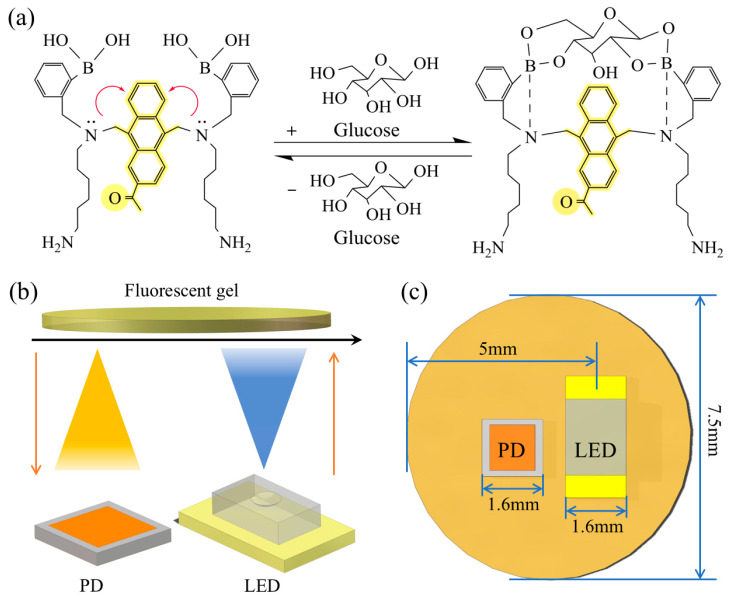
(**a**) Glucose response fluorescence molecular detection principle; (**b**) Design of a fluorescent glucose sensor; (**c**) Top view of a fluorescent glucose sensor.

Boronic acid-based molecules have received much attention in the study of glucose-responsive fluorescent molecules due to their more tunable structural properties [16]. The detection principle is shown in Figure 1a. The boronic acid-based glucose-responsive fluorescent molecules mainly consist of an anthracene group (middle part) and two phenylboronic acid (two side parts) molecular structures. In a glucose-free environment, the fluorescent molecule is in a free state, at which time the molecule can only emit weak fluorescence. In the glucose environment, the dihydroxyl group of the glucose molecule can combine with two phenylboronic acids to form a cyclic boronic ester. This prevents the lone-pair electron transfer of the amine and allows the anthracene group to fluoresce [14]. Therefore, a higher concentration of glucose molecules in the environment will result in a higher fluorescence intensity for the whole system. Thus, the glucose concentration can be characterized by measuring the fluorescence intensity. A gel material is used as a carrier and loaded with boronic acid-based glucose-responsive fluorescent molecules. This approach allows the construction of stable fluorescent glucose sensors. In this study, a dual-component hydrogel network was formed by crosslinking 4-arm polyethylene glycol-N-hydroxysuccinimide ester (TS-PEG) with 4-arm PEG-Amine (TA-PEG) under ambient conditions. The NHS ester groups of TS-PEG react with the amine groups of TA-PEG at room temperature to form stable amide bonds. The entanglement of these long-chain polymers enhances the stability of the hydrogel [17]. Additionally, the amine groups in the fluorescent molecules can also react with the NHS ester groups to form amide bonds, allowing the hydrogel system to immobilize glucose-responsive fluorescent molecules effectively. This hydrogel system has promising applications in the fields of biosensing and fluorescence detection. Compared with conventional glucose sensors, glucose sensors based on fluorescence detection have the advantages of high selectivity for glucose [18], good stability [19,20,21,22], and the possibility of prolonged monitoring [17,23]. It is suitable for fully implantable studies. Therefore, we developed a glucose-responsive fluorescent gel as a sensitive material and designed a fluorescent glucose sensor based on this material.

The signal acquisition of the fluorescent gel is primarily achieved through the optical paths shown in Figure 1b,c, where the center of the Light Emitting Diode (LED) is fixed at a position 5 mm from the left end of the disk. In the energized state, the LED emits light at a wavelength of 450 nm. When the light hits the fluorescent gel, it excites the responsive fluorescent molecules to produce fluorescence at a wavelength of 490 nm. When the excited fluorescence hits the photodiode (PD), the PD can convert it into an electrical signal for output. Therefore, the receiving position of the PD is critical in determining whether the signal acquisition is of high quality. We used Monte Carlo (MC) simulations to study the movement of light through the fluorescent gel layer and the bottom reflected light intensity distribution. They also simulate the deformation of the device after implantation into the subcutaneous tissue by varying the characteristics of the incident light. This includes changes in the incident angle deflection range and emission distance. The optimal receiving position of the PD is designed for the effect of different variables on the distribution of reflected light. Finally, we designed and fabricated fluorescent glucose capsule sensors. We used the sensor to test the glucose level in the subcutaneous tissue of rabbits before and after feeding, as well as to monitor the concentration of glucose solution in real time.

## 2. Methods

### 2.1. Monte Carlo Method for Optical Transport Theory

The Monte Carlo method, also known as random sampling techniques or statistical simulation methods, is a numerical computation framework based on probability theory and statistics [24]. This method originated in the mid-20th century and matured with the development of electronic computer technology. Its fundamental concept lies in approximating deterministic problems through random sampling, particularly showing its unique advantages in simulating complex systems involving randomness. The core strength of this method is its ability to handle difficult problems through traditional analytical methods, especially when dealing with high-dimensional and nonlinear problems. By simulating random processes, the MC method can provide probabilistic estimates of system behavior without the need for precise analytical system modeling. In the research of biomedical photonics, the MC method is used to simulate the propagation of light in tissues. Wang et al. [25] proposed a C-language-based MC simulation framework for simulating the propagation of light in multilayered tissues, which has been widely adopted for its efficient computational performance. Additionally, to adapt to the optical simulation of non-uniform media, researchers have developed various MC simulators. For instance, the MATLAB-based vMC and MMC simulators [26,27,28] use voxel and mesh models, respectively, to simulate photon propagation in media. In these simulations, the photon propagation paths are simulated with random step lengths, and by tracing the paths of a large number of photons, researchers can estimate key physical quantities such as diffuse reflectance, transmittance, and absorbance. The development of these simulators has not only advanced optical simulation technology but also provided a powerful tool for research in the field of biomedical photonics.

The absorption and scattering characteristics of light in biological tissues can be described through two main mathematical frameworks: analytical theory and radiative transfer theory. Analytical theory is based on Maxwell’s equations and starts from the wave nature of light, deriving differential equations for related variables to rigorously describe the propagation behavior of light. However, due to the extremely complex derivation process of analytical solutions and the need for detailed dielectric properties of the tissue, the applicability of this method is limited. In contrast, radiative transfer theory does not rely on Maxwell’s equations but directly describes the transmission process of photons in absorbing and scattering media. This theory uses probabilistic methods to describe photon propagation and is suitable for the study of laser interactions with biological tissues, finding widespread application in this field. When simulating the transmission of light in biological tissues, the MC method shows significant advantages. Simulating the random paths of a large number of photons provides a flexible and rigorous solution and is widely regarded as the gold standard for simulating light transmission in tissues [29,30]. MC simulations can handle any complex tissue structures and non-uniform distributions of optical properties, with a clear physical basis that is easy to understand and implement. The optical properties of tissues, including the scattering coefficient μs, absorption coefficient μa, anisotropy factor g, and average refractive index n, are key parameters in describing the propagation of light in tissues. These parameters not only determine the propagation paths of photons in tissues but also affect the energy deposition of photons, the intensity of diffusely reflected light, and the intensity of transmitted light.

**Figure 2:**
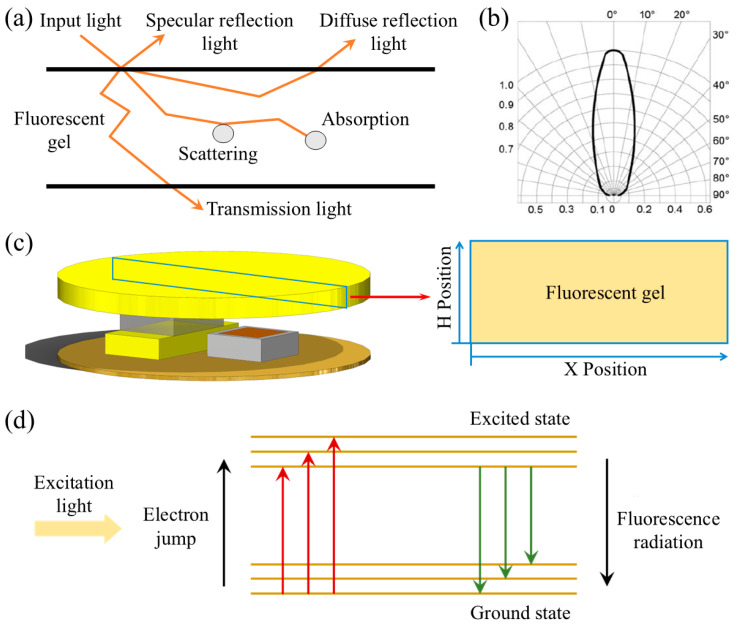
(**a**) Photon propagation in the gel tissue; (**b**) LED emission angle range and light ratio; (**c**) schematic diagram of the simulation area of the intercepted fluorescent gel layer; (**d**) photon excitation process.

The application of the MC method in light transmission theory involves simulating the propagation path of photons in a biological tissue model. The core of this process lies in tracking the random step lengths of each photon within the tissue to simulate the transmission characteristics of light. As shown in Figure 2a, for each emitted photon, a specific initial weight is assigned at the initial stage of its penetration of the tissue model. Subsequently, the step length of the photon is randomly determined based on the optical properties of the tissue, such as the scattering coefficient and the absorption coefficient. When a photon reaches the tissue interface, reflection and transmission phenomena occur due to the refractive index differences between different tissue layers. Reflected photons follow the law of reflection, while transmitted photons continue into the next layer of tissue. Within the tissue, the interaction of photons with microstructures leads to scattering and absorption. Scattering is the process by which the propagation direction of a photon changes randomly after encountering irregular structures in the tissue; absorption is the process by which the energy of a photon is converted into thermal or other forms of energy by the tissue. As the photon approaches the tissue boundary, it may penetrate the boundary or be reflected at the boundary. At the end of each step of the simulation, the energy of the photon decreases according to the absorption probability, and the next step’s step length and scattering angle are randomly sampled according to their respective probability distributions. Photons propagate step by step within the tissue model until they leave the tissue model or are completely absorbed. By emitting a sufficient number of photons and accumulating their path distribution, an accurate approximation of the true solution to the light transmission problem can be obtained. The average contribution of all photon paths can be used to estimate key physical quantities such as diffuse reflectance, transmittance, and absorbance [31].

### 2.2. Fluorescent Gel Layer Tissue Model

In this study, a two-dimensional tissue model was constructed based on the principles of Monte Carlo to maximize the efficiency of receiving reflected light. To achieve this, the PD and LED were configured on the same straight line to ensure direct correspondence of the optical path. Specifically, the area directly above the center of the fluorescent gel layer passing through the straight line was selected as the object of study, as shown in Figure 2c. In the simulation model, the entire model’s dimensions were set to a width of 7.5 mm and a height of 1.9 mm. The fluorescent gel layer occupied a height of 0.8 mm, and there was an air gap of 1.1 mm between the LED emission position and the gel layer. The gel layer was uniformly filled with fluorescent microspheres with a diameter of 10 μm. To simulate the distribution density of fluorescent microspheres, the rectangular gel layer was divided into grid units of equal size. The LED was placed at a horizontal position of 5 mm. To facilitate the tracking of light trajectories, all measurement units in this study were converted to micrometers. Additionally, the emission angle range of the LED was measured, as shown in Figure 2b. During the simulation, the range of incident angles that we focused on was from 75° to 105°. Beams were sequentially injected into the tissue model according to preset incidence parameters. In the process of simulating the propagation of light within the fluorescent gel tissue, the propagation status of the beams, including changes in propagation angle and light intensity, was meticulously recorded. These parameters are crucial for understanding the transmission characteristics of light within the tissue.

When a light beam is incident upon the surface of an object, three fundamental optical phenomena occur, namely, transmission, reflection, and absorption. In this study, the fluorescent gel is modeled as consisting of a pure gel matrix and spherical fluorescent molecules dispersed within it. Upon irradiation of the pure gel, photons primarily undergo anisotropic scattering and transmission processes. Compared to the fluorescent gel, the pure gel absorbs minimal light and thus can be approximated as having an absorption coefficient of zero, exhibiting transparency to light. When the light beam irradiates the fluorescent microspheres, the energy of the photons promotes electrons within the fluorescent molecules to transition from a lower energy level to a higher one, a process known as excitation. Electrons in the excited state are unstable and tend to return to a lower energy level, a process referred to as emission. During the emission process, a portion of the energy is released in the form of light, resulting in fluorescence. The actual reaction process is depicted in Figure 2d. Key parameters of fluorescent molecules include the absorption coefficient and the fluorescence quantum yield. The absorption coefficient describes the ability of fluorescent molecules to absorb light energy under the irradiation of excitation light, while the fluorescence quantum yield quantifies the efficiency with which the molecules convert absorbed energy into fluorescent radiation.

Table 1 demonstrates the parameters used during the simulation. Two parts are included, parameters not related to the deformation of the gel layer such as anisotropy coefficient, absorption coefficient, and scattering coefficient, as well as the parameters related to the deformation, such as the deflection range of the incident angle and the distance between the emission point and the gel layer. The scattering probability can be calculated by the scattering coefficient *μ*:

(1) $$P_{S}=1-e^{-\mu d},$$

where *d* is the motion step and *P_S_* is the scattering probability of the photon. If the scattering occurs, the scattering angle is determined by *s* cos (*μ_s_*), where *μ_s_* and *s* are the anisotropy of scattering and the random number from the group (−1, 1), respectively. In addition, at each step of migration, the light intensity is attenuated by the gel layer and the fluorescent microspheres:

(2) $$I_{\mathrm{new}}=I_{\mathrm{old}}\cdot e^{-\mu_{a}}$$

where the *μ_a_* is the absorption coefficient. Combining scattering and absorption factors, the location and intensity of light can be determined after each movement.

### 2.3. Modeling Methods

**Figure 3:**
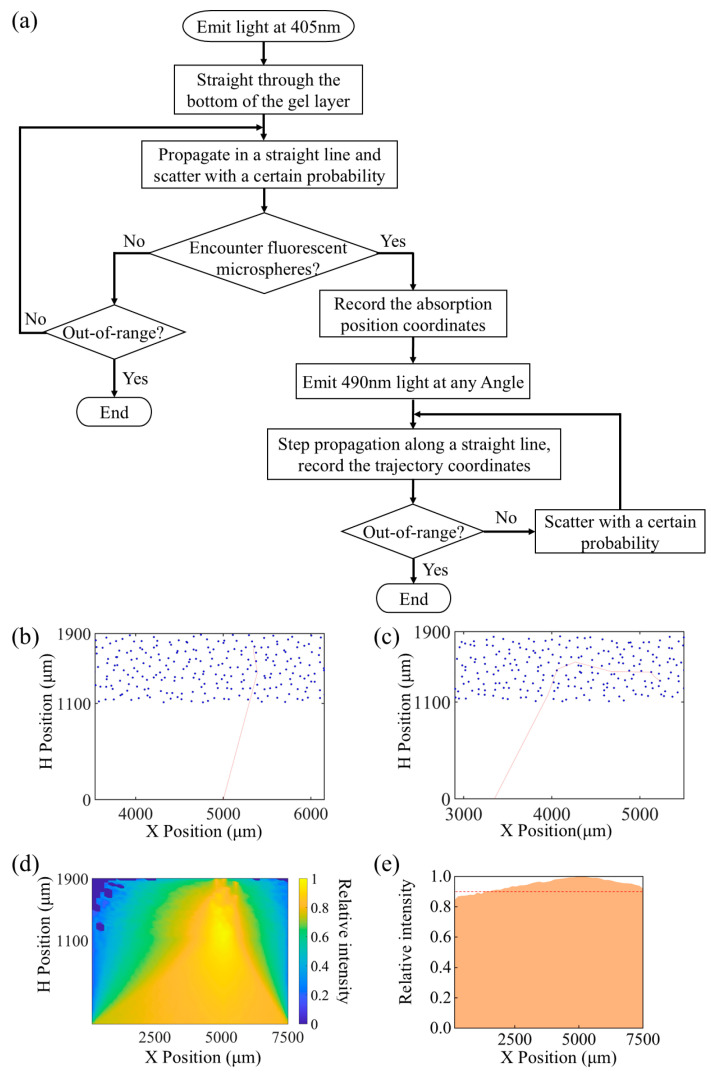
(**a**) Photon transport processes in fluorescent gels based on Monte Carlo methods; (**b**) 405 nm photons encounter fluorescent microspheres to be absorbed process; (**c**) microsphere excitation emits photons of 490 nm wavelength eventually penetrating the process from the bottom of the gel layer (O = 1); (**d**) light intensity cloud of the gel layer and the air layer; (**e**) light intensity distribution in the bottom receiving layer.

We wrote a fluorescence optics simulation program based on the Monte Carlo method. The flow of photon motion is shown in Figure 3a. Initially, a 405 nm wavelength light is emitted sequentially from the LED port according to an angle of 75~105°, based on the measured emission angle range and the corresponding light intensity ratio. We obtained the incident light matrix P by interpolation, which records the incident angle and the corresponding number of light rays. After that, the process of movement of the light was carried out in three steps [32]:

In the first step, the light of the 405 nm wavelength passes through the air layer into the gel tissue at a certain angle of incidence according to the set step size. After that, it propagates according to the step size and refractive index in the gel layer. The light is absorbed when it meets the fluorescent microspheres, and the movement of the light is shown in Figure 3b. The coordinates of the absorbed fluorescent microspheres are recorded in matrix Q.

In the second step, the coordinate positions recorded within the matrix Q are read sequentially. Photons of 490 nm wavelength are emitted at each position read at a randomized emission angle from 0 to 360°. The emitted photons propagate along a straight line. Moreover, the photons have a certain probability of scattering as they propagate through the gel layer and the fluorescent microspheres, thus changing the angle. Eventually, they keep propagating until they are shot out of the gel layer. The motion process is shown in Figure 3c. The horizontal and vertical coordinates of the photon trajectories were recorded into matrices separately, and the trajectory matrices of each photon were integrated into the PX and PY arrays. Additionally, the end positions of the rays are recorded in the O matrix according to the rule of the numbers 1—under the gel to penetrate out to reach the receiving layer; 2—over the gel to penetrate out; and 3—side of the gel to penetrate out.

In the third step, the serial numbers of all the photons ejected from the underside of the gel and the corresponding motion trajectory arrays are extracted from the recorded O matrix. The light intensity clouds propagating in the air and gel layers and the light intensity distribution in the bottom receiving layer are plotted. They are shown in Figure 3d and 3e, respectively. Furthermore, in the graph, the red line marks the contour where the normalized relative light intensity value is 0.9, and the area above the red line represents higher reflected light intensity. The optimal light reception position for the PD can be effectively determined through meticulous analysis of the reflected light intensity distribution and consideration of the high-intensity regions marked by the red line.

## 3. Results and Discussion

The accuracy of fluorescent gel-based glucose sensor devices is sensitive to the intensity of reflected light at the bottom of the gel layer. So, it is necessary to find the maximum point of reflected light intensity thus determining the optimal receiving position of the PD. This is the key to determining whether the signal acquisition is efficient and high quality. We tracked the propagation of the light and recorded the light intensity at each point within the path. The initial intensity of each photon was set to 1. The light intensity at each point on the cloud map depended on the sum of the light intensities at that location and was later normalized. Finally, the light-intensity cloud maps of the gel tissue and the air layer are plotted. In addition, we extracted the relative light intensity distribution of the bottom light-receiving layer and plotted the bottom light intensity distribution. It was used to observe the distribution of reflected light reaching the bottom after penetrating out from underneath the gel layer to better determine the installation position of the PD.

When a fluorescent gel device implanted into the skin receives external stresses such as squeezing or twisting, the device will be affected in receiving light signals. In the subsequent experiments, the molecular density of fluorescent microspheres, the thickness of the hydrogel layer, the distance between the hydrogel layer and the emission position, and the deviation of the incident angle were varied. Cloud maps of light intensity and distribution plots of bottom light intensity were generated to observe the position of the brightest spot in the cloud map. It should also be noted that we regulated the total number of incident light rays at different angles during the simulation. When the increase in the number of light rays is not enough to make a significant change in the light intensity distribution of the cloud map, the simulation results are considered to have reached convergence at this point.

### 3.1. Change the Concentration of Fluorescent Molecules

**Figure 4:**
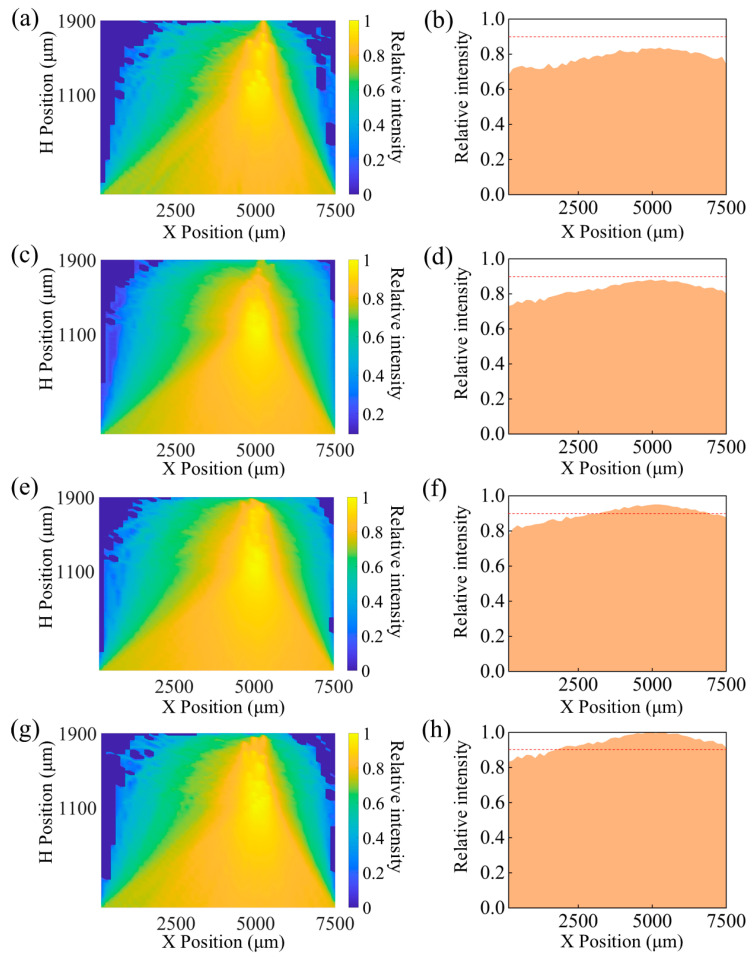
(**a**) Light intensity cloud map and (**b**) bottom light intensity distribution map at a fluorescent molecule concentration of 1.25 mg/mL; (**c**) light intensity cloud map and (**d**) bottom light intensity distribution map at a concentration of 2.5 mg/mL; (**e**) light intensity cloud map and (**f**) bottom light intensity distribution map at a concentration of 5 mg/mL; (**g**) light intensity cloud map and (**h**) bottom light intensity distribution map at a concentration of 10 mg/mL.

The concentration of fluorescent molecules significantly affects the intensity of the excitation light signals received by the PD. The reaction between fluorescent molecules and glucose can be categorized into two scenarios. In the first scenario, when the concentration of fluorescent molecules is higher than that of glucose molecules, all glucose molecules react with fluorescent molecules, generating fluorescent-reflected light intensity. Under these conditions, further increasing the concentration of fluorescent molecules does not affect the resulting reflected light intensity. The second scenario occurs when the concentration of fluorescent molecules is lower than that of glucose molecules, in which case all fluorescent molecules are excited to produce fluorescent-reflected light intensity. Our simulation study primarily focuses on this second scenario and involves a statistical analysis of the fluorescent-reflected light intensity. In the simulation, the initial concentration of fluorescent molecules was set to 5 mg/mL, based on the concentration used in the preparation of the fluorescent gel. Subsequently, by systematically increasing or decreasing the concentration of fluorescent molecules, the impact of these changes on the distribution of the cloud pattern and the bottom light intensity distribution was studied, with the final results presented in Figure 4. Additionally, the ratio of the reflected light intensity at the bottom, generated by fluorescent molecules, to the total emitted light intensity, was statistically analyzed to observe the changes in reflected light intensity following the excitation of fluorescent molecules at different concentrations. The statistical results are detailed in Table 2. The results indicate that a decrease in the concentration of fluorescent molecules leads to a reduction in the light absorbed by the microspheres, which in turn reduces the light reflected to the bottom after excitation of the microspheres, resulting in a decrease in the light intensity received by the PD.

Although the simulation results demonstrate a positive correlation between the increase in fluorescent molecule density and the enhancement of received optical signal strength, in practical sensor applications it is also necessary to consider the interference from the device’s inherent working signals and ambient light intensity. To ensure the accuracy of the measurement results, the received optical signal must surpass the threshold of external signal interference. Shibata et al. [16] designed a glucose sensor based on hydrogel microspheres, revealing that the boronic acid part of the fluorescent molecules has an extremely high selectivity for glucose. Moreover, the structure of the gel allows for better contact between fluorescent molecules and glucose molecules, thus being more conducive to the sensor producing fluorescence. In this study, the concentration of fluorescent molecules used was 5 mg/mL, which was sufficient to support the continuous detection of glucose concentration in vivo. Additionally, subsequent animal experimental results indicate that fluorescent molecules at this concentration exhibited excellent fluorescent response characteristics. Studying the impact of the number of fluorescent molecules is of great significance in optimizing sensor performance and ensuring monitoring accuracy.

### 3.2. Change the Thickness of the Tissue Layer

#### 3.2.1. Change the Thickness of the Gel Layer

**Figure 5:**
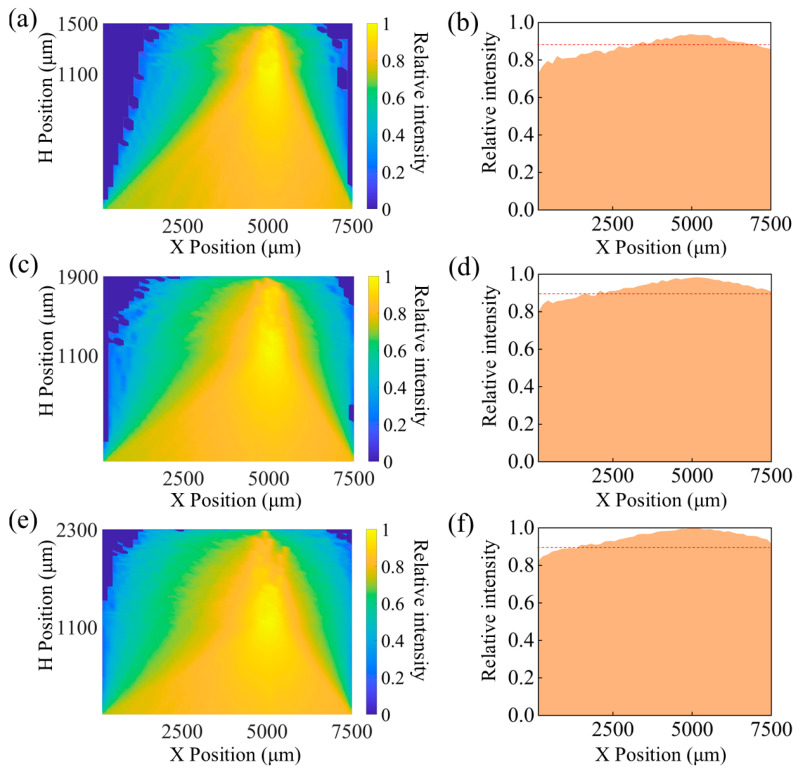
(**a**) Optical intensity contour plot and (**b**) bottom optical intensity distribution plot for a gel thickness of h = 0.4 mm; (**c**) optical intensity contour plot and (**d**) bottom optical intensity distribution plot for a gel thickness of h = 0.8 mm; (**e**) optical intensity contour plot and (**f**) bottom optical intensity distribution plot for a gel thickness of h = 1.2 mm.

In the simulation study, it was necessary to consider the impact of varying thicknesses of different tissue layers on the simulation results. The fluorescent molecule density was maintained at 5 mg/mL, with all other variables held constant. Two sets of simulation experiments were conducted: one varying the thickness of the gel layer and the other altering the distance between the LED and the gel layer. Initially, a simulation analysis was performed to assess the effects of changes in the gel layer thickness. During the fabrication of the gel layer, process errors may lead to inconsistencies in the thickness of the gel layer, thereby affecting the distribution of light intensity at the bottom. In the simulation, the thickness of the gel layer was adjusted to 0.4 mm, 0.8 mm, and 1.2 mm. The corresponding light intensity distributions were recorded and the results are detailed in Figure 5a–f. Furthermore, the ratio of the light intensity reflected from the bottom, generated by fluorescent molecules, to the total emitted light intensity was statistically analyzed. The results are presented in Table 3. Simulation results indicate that, under the premise of equal fluorescent molecule density, increasing the thickness of the gel layer can enhance the light intensity received at the bottom. However, in the actual preparation of the gel layer, the thickness often exceeded 0.6 mm, and to ensure the accuracy of the measurements, we prioritized selecting gel layers that were uniformly thick and full for subsequent measurement work. Therefore, we chose a gel with a thickness of 0.8 mm as the baseline to conduct simulation experiments to determine the optimal installation position for the PD. Additionally, subsequent in vivo measurement experiments confirmed that using a gel layer with a thickness of 0.8 mm can achieve accurate monitoring of glucose concentration.

#### 3.2.2. Change the Distance Between the LED and the Gel Layer

**Figure 6:**
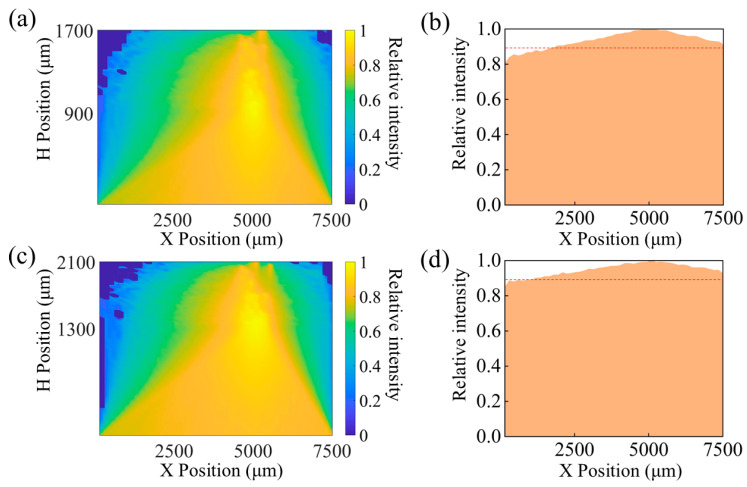
(**a**) Optical intensity contour plot and (**b**) bottom optical intensity distribution plot when the distance s*s* between the emission plane and the gel layer plane is s = 0.9 mm; (**c**) optical intensity contour plot and (**d**) bottom optical intensity distribution plot when the distance s*s* is s = 1.3 mm.

Similarly, due to the deformation of the gel layer, the distance between the emission plane and the plane of the gel layer may change thereby changing the bottom light intensity distribution. Here, we varied the distance between the LED emission plane and the gel layer plane to 0.9 mm and 1.3 mm (the setting between the original devices was 1.1 mm). Figure 6a,b show the light intensity cloud and the distribution of light intensity at the bottom for the case of distance 0.9 mm, and Figure 6c,d for the case of distance 1.3 mm. The results show that the further the distance between the emission plane and the gel layer plane, the greater the light intensity at the bottom receiving layer edge. However, this did not affect the overall distribution trend and had little effect on the optimal receiving position.

### 3.3. Change the Direction of Deflection of the Angle of Incidence

**Figure 7:**
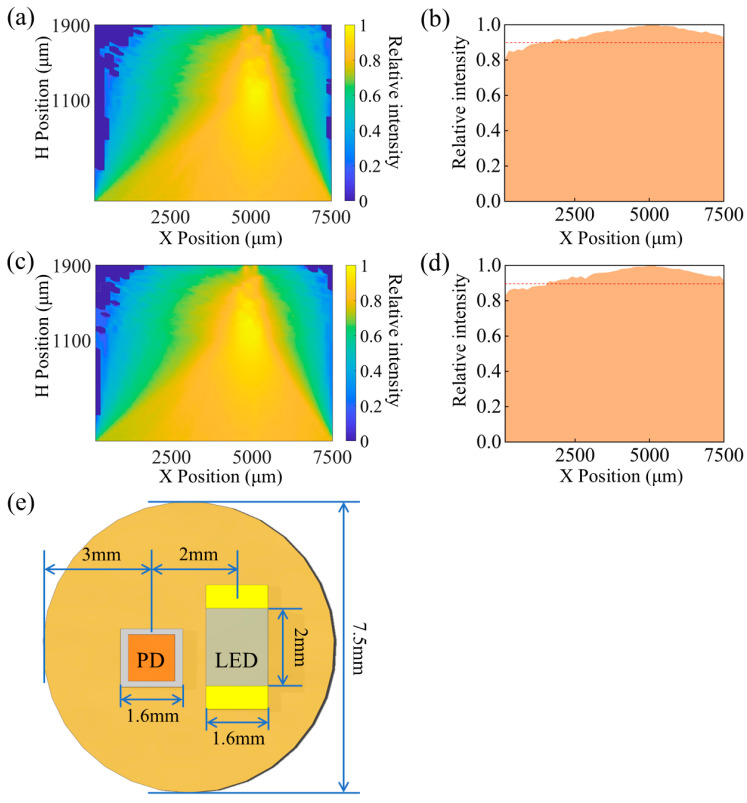
(**a**) Light intensity cloud and (**b**) bottom light intensity distribution in the range of incident angle 70~100°; (**c**) light intensity cloud and (**d**) bottom light intensity distribution in the range of incident angle 80~110°; (**e**) fluorescent glucose sensor transmit/receive position.

When the fluorescent gel device inside the skin is subjected to external stress, it can cause the device to be affected in receiving the incident light signal. Here, we keep the incidence angle range of 30° and deflect the original 75~105° incidence cases. The deflected incident angle cases are divided into two groups, 70~100° and 80~110°. The results after the simulation are shown in Figure 6a–d. Figure 7a,b show the light intensity cloud and bottom light intensity distribution for the case of incidence angle range 70~100°. Figure 7c,d show the case of incidence angle range 80~110°. The cloud map results of the two simulations do not show a large difference. This indicates the deflection of the incidence angle on the bottom light intensity distribution. That is, it has almost no effect on the optimal receiving position of the PD.

Taking into account the performance parameters of existing components and the dimensions of the packaged LED and PD, the center of the PD was positioned 3 mm from the left end of the disk, as shown in Figure 7e. Subsequent system-level testing experiments will demonstrate the sensor’s ability to detect changes in fluorescence intensity caused by variations in glucose concentration.

### 3.4. Design of Fluorescent Glucose Capsule Sensor

**Figure 8:**
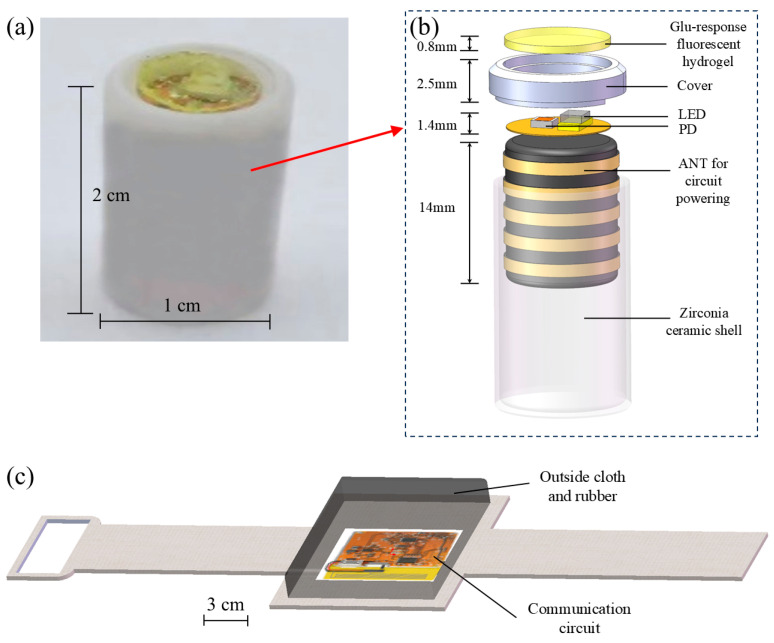
(**a**) Physical fluorescent glucose capsule sensor; (**b**) schematic diagram of glucose capsule sensor structure; (**c**) schematic diagram of wearable armband structure.

Based on the above simulation analysis, we designed and fabricated a subcutaneous, fully implantable wireless battery-free capsule system. It can be used to measure glucose concentration in real time. The whole system is divided into two parts: the glucose capsule sensor (Figure 8a) and the wearable armband (Figure 8c). Figure 8b shows the schematic structure of the glucose capsule sensor. From top to bottom are the fluorescent gel layer, the ceramic cover plate for encapsulation, the signal transmitting and receiving circuits (mainly including LEDs and PDs), the internal coil ANT used to power the LEDs and transmit information, and the zirconia ceramic housing. All assembly interfaces were glued using a waterproof medical UV adhesive. The integrated glucose capsule sensor is 1 cm in diameter and 2 cm in height, making it suitable for minimally invasive implantation. Figure 7c shows the wearable armband structure. It is mainly divided into an external plastic shell, a fabric and rubber blend, and an internal communication circuit.

To use it, the capsule is first implanted into the subcutaneous tissue through minimally invasive surgery. The gel reacts with glucose in the tissue and fluoresces in the presence of an LED, which receives optical information from the PD to generate a sensing signal. A wearable armband is attached to the capsule at the implantation site during measurement. The communication circuitry of the armband can connect to the capsule via Near Field Communication (NFC) technology. This enables wireless data transmission and power transfer functions. The received data are then transferred to a customized mobile app via Bluetooth. Thus, continuous monitoring and visualization of glucose are performed.

### 3.5. Animal Experiments and Solution Calibration Tests

To demonstrate this subcutaneous, fully implantable wireless battery-free capsule system, we performed tissue glucose level tests in rabbits before and after feeding, respectively, and real-time glucose solution concentration monitoring experiments.

**Figure 9:**
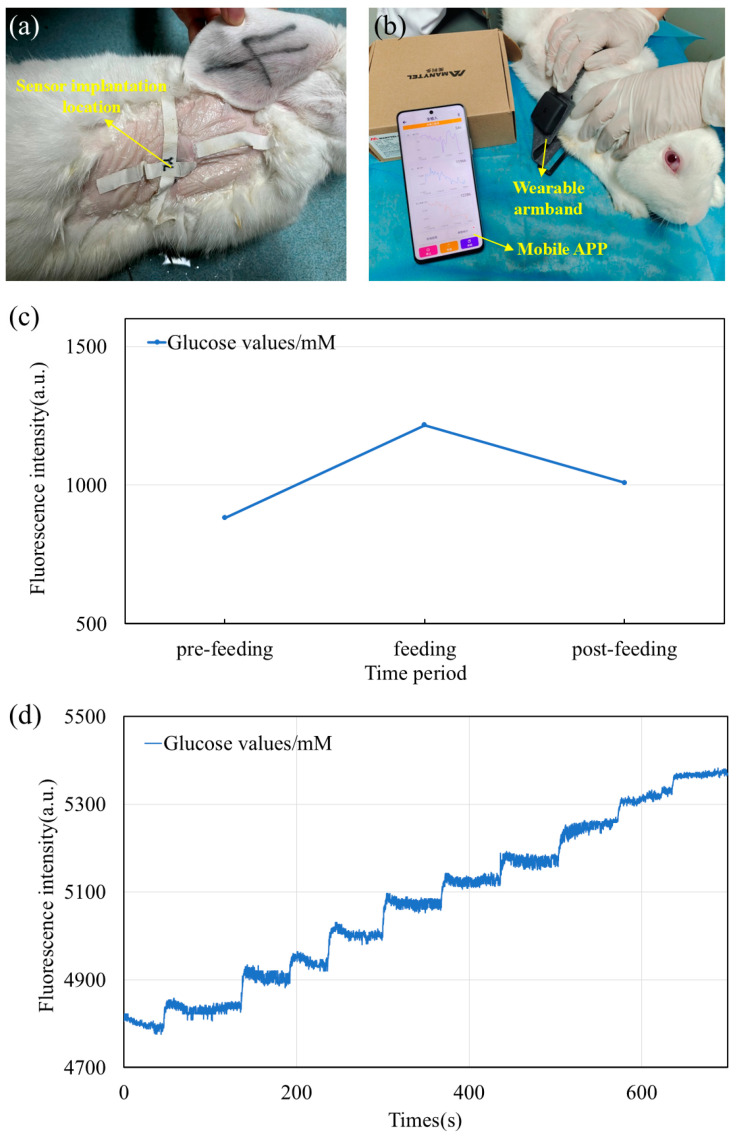
(**a**) Fluorescent glucose capsule sensor implanted on the back of a rabbit. (**b**) Wearable armband strapped to the back of a rabbit to receive signals in real time using a cell phone app. (**c**) Values of glucose level changes in the subcutaneous tissue of a rabbit before and after feeding. (**d**) Real-time measurement of glucose level for a changing concentration of glucose solution.

The living rabbit experiment was performed at the Zhejiang Center of Laboratory Animals (ZJCLA, Hangzhou, China) and approved by the Institution Animal Care and Use Committee (IACUC) of the center (ZJCLA-IACUC-20010365). We anesthetized the rabbit using an anesthetic and shaved its back. The glucose capsule sensor was then implanted into the subcutaneous tissue of the rabbit’s back through minimally invasive surgery. This is shown in Figure 9a. After waiting for the rabbit to wake up and be able to move on its own, a wearable armband was attached to the rabbit (Figure 9b). The average glucose level of the rabbit during this time was measured on a customized mobile app through a signaling device. The rabbit was fed after some time. The amount of change in the glucose level of the rabbit during the feeding period was observed and the data were recorded. Finally, we waited for a while after the feeding was over And measured the glucose level of the rabbits again. The results of the experiment are shown in Figure 9c. The results showed that there was a significant increase in the glucose level of the tissues of rabbits during the feeding time. After ending the feeding for a while, it dropped to be almost the same as the pre-feeding level, which is consistent with the actual situation.

For real-time glucose solution concentration monitoring experiments, the whole testing process needs to be carried out in a black box to avoid ambient light interference. We first immersed the glucose capsule sensor in a sample box containing glucose solution. Meanwhile, a wearable armband with an NFC coil was placed under the box at the capsule location for wireless signal transmission. The waveform curve of glucose concentration in the solution was read using the app after powering on. After waiting for the waveform curve to stabilize over time, a glucose solution with a concentration of 0.4 mM was dropped into the solution. After that, continuous magnetic stirring was used to make the solution mix well. After waiting for the waveform curve to stabilize, another drop of glucose solution with a concentration of 0.8 mM was added to the solution, and so on to observe the change in the waveform curve. Each time, the concentration of glucose solution was increased by 0.4 mM compared to the previous one, and magnetic stirring was used to make the solution mix well after dropping to reach the reaction concentration. The final waveform change curve situation is shown in Figure 9d. The results of the measured glucose level curves show that the fluorescent glucose sensor can monitor the glucose concentration in solution accurately and with clear signals in real time.

## 4. Conclusions

In this paper, we explored a glucose sensor for continuous glucose monitoring based on minimally invasive implantation of fluorescent glucose capsule sensor. In order to optimize the fluorescent glucose capsule sensor structure design, a two-dimensional model was created to simulate the movement of light in the gel and air layers inside the sensor. The optimal receiving position of the photoelectric sensor PD was designed by mapping the light-intensity cloud. Additionally, the experiments involved altering the density of fluorescent molecules, varying the thickness of tissue layers, and adjusting the deflection angle of the incident light to observe whether the reception position would be affected. Finally, we designed and fabricated a subcutaneous, fully implantable fluorescent glucose capsule sensor system. Subcutaneous tissue glucose levels were tested before and after feeding in rabbits, respectively, as well as real-time monitoring of glucose solution concentration. The monitoring results showed the advantages of high accuracy on glucose, good stability, and the ability to be monitored for a long time. This opens up a new approach to the design of glucose sensors and the measurement and control of glucose levels. Overall, this study provides a robust foundation for the fluorescent glucose capsule sensor design and offers promising directions for future real-time monitoring of glucose research aimed at self-monitoring blood glucose, ultimately contributing to prevent diabetic complications of the heart, kidneys, retina, and nervous system. This will enable future research to continue the advancement of minimally invasive blood glucose monitoring sensors.

## Figures and Tables

**Table 1:** Parameters used in the MC simulation.

Quantity	Symbol	Value	Unit
Tissue absorption coefficient	$\mu_{\mathrm{at}}$	0.001	${\mathrm{cm}}^{-1}$
Micro-balloon absorption coefficient (405 nm)	$\mu_{\mathrm{mt}}$	41	${\mathrm{cm}}^{-1}$
Micro-balloon absorption coefficient (490 nm)	$\mu_{\mathrm{mt}}$	4.1	${\mathrm{cm}}^{-1}$
Tissue anisotropy of scattering	$\mu_{\mathrm{sat}}$	0.8	
Micro-balloon anisotropy of scattering	$\mu_{\mathrm{sam}}$	0.2	
Tissue scattering coefficient	$\mu_{\mathrm{st}}$	10.3	${\mathrm{cm}}^{-1}$
Micro-balloon scattering coefficient	$\mu_{\mathrm{sm}}$	80	${\mathrm{cm}}^{-1}$
Light incident angle deflection	θ	75–105	degree
		70–100	degree
		80–110	degree
Gel layer thickness	*h*	0.4–1.2	mm
Distance between launch position and gel layer	s	0.9–1.3	mm

**Table 2:** Effect of varying the density of fluorescent molecules on fluorescent-reflected intensity.

Fluorescent Molecular Density	Fluorescent Reflection Intensity/Total Intensity (%)
1.25 mg/mL	7.73
2.5 mg/mL	12.2
5 mg/mL	24.64
10 mg/mL	39.84

**Table 3:** Effect of varying gel layer thickness on fluorescent reflection intensity.

Gel Layer Thickness (mm)	Fluorescent Reflection Intensity/Total Intensity (%)
0.4	14.37
0.8	24.64
1.2	30.46

## Data Availability

Data are contained within the article.
